# BEP-IM: A Vehicular Crowdsensing Incentive Mechanism to Drive Sustained Spatial Coverage and Proactive Sensing Shaping

**DOI:** 10.3390/e28050499

**Published:** 2026-04-28

**Authors:** Jiamin Zhang, Lisha Shuai, Jiuling Dong, Gaoya Dong, Xiaolong Yang, Keping Long

**Affiliations:** School of Computer and Communication Engineering, University of Science and Technology Beijing, Beijing 100083, China; zhangjm_ustb@163.com (J.Z.); promethusls@outlook.com (L.S.); hndongjiuling@163.com (J.D.); gaoyadong@ustb.edu.cn (G.D.); longkeping@ustb.edu.cn (K.L.)

**Keywords:** vehicular crowdsensing, reference-dependent, loss aversion, operant conditioning, incentive mechanism

## Abstract

In the Internet of Vehicles, vehicular crowdsensing is crucial for alleviating traffic congestion and ensuring the safety of autonomous driving. However, practical vehicular crowdsensing processes face dual challenges of skewed spatial distributions of vehicles and inadequate data quality guidance. These issues cause sensing redundancy in high-participation areas (HPAs) and coverage deficits in low-participation areas (LPAs), while also leading to unstable data quality. Given that participants’ decisions are profoundly influenced by bounded rationality and psychological preferences, this paper proposes a collaborative incentive mechanism integrating behavioral economics and psychology (BEP-IM) to drive sustained spatial coverage and proactive sensing shaping. First, to mitigate coverage deficits in LPA, a reference-dependent two-sided selection and bidding strategy (RD-TSB) is designed to guide participants toward LPA via a reference-driven utility evaluation. Concurrently, a loss-aversion-based sustained incentive strategy (LA-RPI) is introduced to enhance their sustained participation within LPAs by amplifying loss perception. Furthermore, to overcome weak data quality constraints, an operant conditioning-based proactive sensing shaping strategy (OC-SFQ) is constructed, utilizing a closed-loop mechanism of relative improvement, variable-ratio reinforcement, and association updating to drive participants to output high-quality data. Simulation results demonstrate that the proposed mechanism effectively increases participation frequency in LPAs and optimizes sensing data quality.

## 1. Introduction

With the rapid proliferation of high-level autonomous driving and smart city infrastructure [1], the Internet of Vehicles (IoV) has emerged as the foundational architecture for real-time traffic state assessments and collaborative decision-making [2]. Precise perception of the urban traffic environment is no longer merely an auxiliary function; rather, it is a prerequisite for alleviating large-scale traffic congestion [3] and ensuring the safety of autonomous vehicle fleets in critical tasks [4]. By leveraging the pervasive mobility and multimodal sensing capabilities of connected vehicles, vehicular crowdsensing (VCS) provides a high-resolution, cost-effective paradigm for acquiring dynamic urban data that traditional fixed infrastructure cannot capture [5].

However, the practical deployment of VCS tasks remains severely constrained by two intertwined bottlenecks: the uneven spatial distribution of participants and the inadequate guidance for data quality [6]. First, the inherent coupling between sensing coverage and vehicle locations induces a geographical Matthew effect. Many vehicular participants naturally aggregate in high-participation areas (HPAs) to minimize fuel consumption and travel expenses. This aggregation leaves peripheral roads and critical low-participation areas (LPAs) in sensing blind spots [7,8]. In this paper, HPA and LPA refer to regions with systematically different local participation density and sensing supply-demand conditions. Specifically, HPA denotes regions where participant density is relatively high and the local sensing supply can generally satisfy task demand, whereas LPA denotes regions where participant density is relatively low and the local sensing supply is insufficient relative to the task demand, making these regions more vulnerable to task backlog and coverage deficits. In practical vehicular crowdsensing scenarios, an HPA typically corresponds to urban centers, arterial roads, major intersections, or hotspot road segments during peak periods, while an LPA more often corresponds to peripheral roads, suburban branch roads, remote road segments, or off-peak road areas. More detailed operational clarification and quantitative criterion for HPAs/LPAs are provided in Appendix A. Second, existing incentive mechanisms frequently prioritize the quantity of participation over the intrinsic value of the data. Without effective behavioral guidance and quality-based reinforcement, participants tend to adopt low-effort strategies to minimize their costs. This tendency results in highly variable data reliability [9], which fails to meet the stringent precision requirements of safety-critical IoV applications [10]. Consequently, bridging the gap between the randomness of participant behavior and the deterministic demand for high-quality, full-domain sensing remains an urgent open problem.

To address these challenges, existing research on incentive mechanisms generally follows three main paradigms. The first paradigm involves differential incentivization, which is guided by coverage gaps or target distributions. This approach configures higher rewards for LPAs and reduces the attractiveness of HPAs to form differential price signals, aiming to steer participants toward areas with weak sensing coverage. The second paradigm focuses on linking scheduling with payments to compensate for detour costs. This method explicitly considers the opportunity costs incurred when vehicles deviate from their original routes. It typically employs reverse auction or combinatorial auction mechanisms to compensate for mobility costs while ensuring truthfulness and individual rationality, thereby encouraging participants to travel to LPAs under guaranteed rules. The third paradigm relies on adaptive long-term regulation driven by behavior and learning. This approach treats participants as decision-makers capable of comparison and learning. By incorporating behavioral preferences and data quality into dynamic utility functions, it utilizes learning or equilibrium mechanisms to continuously adjust strategies, thus maintaining participant motivation in uncertain environments.

Despite the effectiveness of these existing studies, several limitations persist. Many models assume that participants are strictly rational, neglecting the influence of complex psychological factors on their decision-making processes. Furthermore, coverage incentives and quality incentives are frequently treated in isolation. While increased compensation might boost participation rates, it does not necessarily guarantee high-quality contributions and can even induce speculative reporting. Additionally, collaborative sensing in the IoV exhibits a distinct multi-round interactive nature. Elevating rewards in a single round struggle to form stable behavioral constraints, often resulting in short-term responses, long-term decay, and strategic price-chasing behaviors. Therefore, the prevailing paradigm of using price increases to attract participants still exhibits structural gaps in explaining actual participant decisions, balancing coverage with quality, and ensuring long-term stability.

To overcome these deficiencies, this paper proposes a collaborative incentive mechanism that integrates behavioral economics and behavioral psychology, termed BEP-IM, to jointly optimize sensing performance across both spatial and quality dimensions. The primary contributions are outlined in three aspects.

1. At the spatial level, the proposed mechanism introduces effects from behavioral economics, including the reference effect and loss aversion. It constrains bidding behaviors through a reference-driven utility evaluation to effectively guide participants from HPAs to LPAs. It also reinforces the psychological effect of perceiving a failure to meet targets as a loss through the asymmetric modeling of gains and losses, which significantly enhances participant stickiness in LPAs.
2. At the quality level, the mechanism incorporates operant conditioning and shaping concepts from behavioral psychology. It constructs a proactive sensing behavior-shaping module based on variable-ratio reinforcement and dynamic association updating mechanisms. This module uses intermittent reinforcement rewards to guide participants to progressively increase their effort levels, thereby forming a stable habit of contributing high-quality data.
3. Finally, simulation results demonstrate that the proposed scheme yields improvements in behavioral migration and proactive sensing shaping.

The remainder of this paper is organized as follows. Section 2 and Section 3 introduce the related work and theoretical foundations, respectively. Section 4 details the proposed incentive mechanism and algorithm design. Section 5 validates the effectiveness of the scheme through comparative experiments and systematically discusses the results. Section 6 concludes the paper and outlines future research visions.

## 2. Related Work

Existing studies generally recognize that the spatial clustering of vehicle mobility in the Internet of Vehicles leads to persistent coverage gaps in LPAs. To address this imbalance in spatial sensing, the literature gradually develops three representative research directions centered on guiding participants to move to LPAs and execute tasks. These directions include differential incentives driven by coverage gaps and target distribution, scheduling-payment joint incentives driven by cost compensation, and adaptive incentives driven by behavior and quality.

The first direction can be summarized as making LPAs more rewarding. Its core idea is to use coverage gaps or target distribution as guidance and generate differentiated price signals by assigning higher rewards to LPAs and reducing the relative attractiveness of hotspot areas, thereby steering participants from congested areas to LPAs and improving overall coverage efficiency. For example, ref. [11] constructs a reverse auction framework with a reserve price. According to supply and demand conditions, the platform gradually lowers the reserve price of tasks in HPAs across rounds, thereby shifting participants’ bidding interest, at the level of market signaling, toward tasks or areas with insufficient participants and improving the completion rate in LPAs. They also use critical payment to ensure truthful bidding. Ref. [12] focuses on compensation for location diversity. A vehicle’s payoff is related to the spatial diversity of its route, and rewards are higher in LPAs; that is, vehicle utility is inversely related to traffic density in the current area. As a result, vehicles are encouraged to deviate from crowded routes and move toward isolated places. This process is modeled as a non-cooperative game, and its stable solution is a Nash equilibrium. Ref. [13] models platform demand as a target spatiotemporal distribution and aims to minimize the gap between the sampling distribution and the target distribution, following a distribution-matching idea like KL divergence. Under budget constraint, it selects the vehicles to be incentivized, and their candidate movement plans and then determines the corresponding compensation. Since LPAs yield higher marginal coverage gains, they are assigned a stronger incentive intensity, which enables targeted supplementation. Although such methods are intuitive and easy to deploy, they usually assume that participants are rational economic agents who respond immediately to price signals. As a result, they give relatively limited consideration to differences in opportunity cost, behavioral preference, and long-term repeated participation. Therefore, under dynamic supply–demand conditions and budget constraints, these methods may suffer from declining incentive efficiency or unstable coverage improvement.

The second direction can be summarized as explicitly accounting for the detour cost and using mechanism rules to ensure that participants are willing to move to LPAs. This line of work incorporates the cost caused by route deviation into mechanism design in an explicit manner. It usually adopts reverse auctions or combinatorial auctions and jointly determines task allocation and payment computation. Under the requirements of truthfulness and individual rationality, the mechanism compensates for the mobility cost, so that participants are willing to incur additional travel to complete tasks in LPAs. For example, ref. [14] proposes a mobility-based incentive mechanism, which compensates participants for the extra cost of visiting LPAs to balance spatiotemporal coverage and achieve a win–win outcome between platform data diversity and participant utility. To address the challenge of incomplete trajectory information in online settings, ref. [15] jointly designs trajectory scheduling and incentive payment. By using an auction mechanism to characterize the sensing cost and detour cost, the platform offers higher marginal compensation for tasks in sparse spatiotemporal regions, thereby ensuring that drivers are willing to accept scheduling decisions when the expected return covers the detour loss. In this way, the mechanism promotes vehicle coverage in spatiotemporal regions that are otherwise difficult to cover. The main advantage of this class of methods is that it can handle strategic bidding by participants and provide provable mechanism guarantees. However, such methods usually rely on stronger assumptions about the information structure and incur higher computation and communication overhead. Moreover, they mainly focus on inducing participants to execute tasks, while paying relatively limited attention to the endogenous improvement in data quality and the shaping of long-term behavior.

The third direction can be summarized as treating participants as decision-makers who compare, learn, and adapt over time. Its core idea is to incorporate opportunity cost, behavioral preference, and data quality into dynamic utility and constraint design and then continuously adjust incentives through adaptive reward and penalty schemes, learning mechanisms, or equilibrium-based control. In this way, participants remain willing to move to LPAs and submit high-quality data even under uncertainty. For example, ref. [16] designs a behavioral–economics-based incentive mechanism for the problem of uneven participant distribution. By modeling participants’ behavioral tendencies, their method achieves a more balanced participation pattern. Ref. [17] proposes a scheme that induces vehicles to deviate from their planned routes and visit the PsI through reward design. In their method, the reward coefficient is associated with the time interval since the last sampling event. When the interval is large, the coefficient approaches 1, whereas when the interval is small, it approaches 0. As a result, self-interested vehicles choose the PsI that improves temporal coverage to maximize its utility. Although the primary objective is temporal coverage, the mechanism also encourages vehicles to visit the off-route PsI, which in turn produces an additional gain in spatial coverage. Ref. [18] proposes a voting-based incentive model. First, participants are selected through PDAs, which follows the reverse-auction idea and jointly considers bids, execution capability, and the number of valid tasks. Second, BBER is used to achieve budget balance and provide extra rewards. Third, EA, namely a voting mechanism, evaluates both the credibility of task results and individual completion performance, based on which reward and penalty payments are determined. Its support for moving to LPAs is indirect. More specifically, it is better suited to tasks specified by location and time, and it improves the credibility and controllability of task execution, including tasks at remote or sparse locations, by strengthening the link between completion performance, credibility, and reward or penalty. Ref. [19] proposes a probabilistic incentive mechanism modeled as a two-stage non-cooperative game with a macro stage and a micro stage. In the macro stage, the expected utility of visiting each POI is estimated by considering the potential behavior of other vehicles and the global traffic state. In the micro stage, vehicles compete for reward sharing within the same POI. Vehicles then choose routes that deviate from their planned trajectories and maximize expected utility. The resulting trajectory Nash equilibrium improves coverage in LPAs. In addition, ref. [20] adopts the Gigwalk idea that tasks receive a higher price when they remain unaccepted for a long time and assign higher rewards to untrodden segments. At the same time, it considers the opportunity cost of drivers whose main income comes from passenger transport and designs a task-allocation strategy that jointly accounts for short-term and long-term returns, so that drivers are willing to move toward low-coverage road segments under an acceptable payoff trade-off. Ref. [21] proposes Environment Cost (EC) to quantify actual effort and designs a spatiotemporally fair incentive mechanism based on the Jain index, so that tasks in LPAs become acceptable in terms of the net payoff. It also introduces a comparison between MECT and EC to detect speculative behavior and prevent the incentive mechanism from being exploited, thereby stably promoting task execution in LPAs. Ref. [22] is the first to introduce willingness to traverse to describe regional remoteness and proposes a two-stage mechanism. On the vehicle side, multi-agent deep reinforcement learning is used to jointly learn route adjustment and bidding. On the platform side, task allocation and compensation are further adjusted according to the reported routes and bids, so that vehicles are encouraged, under a limited budget, to actively detour through remote areas and fill coverage gaps. Ref. [23] proposes ASQ, which unifies coverage and reliability into a single QoI measure and further develops Mutually Assisted Belief-aware Vehicle Dispatching to estimate reliability under uncertainty and allocate monetary incentives. Experimental visualization shows that the system dispatches vehicles from HPAs to LPAs, while prioritizing vehicles with higher net reliability to improve the error performance in low-coverage regions. There are also studies such as [24], adopting a cost-avoidance strategy in which inference is used to replace part of the actual sensing effort in LPAs. Rather than directly pushing participants to LPAs through high rewards, these methods reduce dependence on physical sensing in LPAs by combining selective recruitment with inference-based completion. Overall, this line of workplaces greater emphasis on the coordination among coverage, quality, and behavioral feedback, but it also introduces more complex model design challenges.

Compared with traditional incentive mechanisms, the distinction of the mechanism proposed in this paper lies not only in its specific optimization form, but more importantly in its modeling perspective and regulation objectives. Existing methods, whether based on reverse auctions, combinatorial auctions, or deep-learning-assisted auction optimization, still mainly focus on task allocation, payment pricing, truthfulness constraints, or resource-utilization efficiency. In essence, they regulate participants’ one-shot decision-making behavior through external monetary rewards. By contrast, this paper further models’ participants as boundedly rational decision-makers with dynamic behavioral feedback, and incorporates reference dependence, loss aversion, and operant conditioning to construct a cross-round closed-loop incentive process from three dimensions: spatial guidance, sustained participation, and quality shaping. Therefore, the objective of this paper is no longer limited to one-shot allocation efficiency or local payoff optimality but instead places greater emphasis on achieving the long-term stable guidance of participation behavior and the sustained formation of high-quality data output in repeated interaction scenarios.

While existing studies have mitigated the coverage deficit in LPAs through differential pricing, mechanism design, and adaptive incentives, two critical limitations remain. First, reference effect mechanisms based on behavioral economics typically define the reference point as a static constant. This rigid configuration struggles to adaptively adjust to the dynamic evolution of the supply and demand gap in LPAs, frequently causing delayed incentive signals and resource misallocation under rapid spatiotemporal demand fluctuations. Second, current quality incentives predominantly focus on increasing participant payoffs in exchange for higher quality. They rarely establish an actionable reinforcement feedback loop from the perspective of behavior shaping. Consequently, these approaches fail to continuously enhance data quality without significantly inflating the cumulative utility of participants.

## 3. Theoretical Foundations

1. Reference effect

The reference effect is one of the core theories in behavioral economics for characterizing boundedly rational decision-making [25]. Prior studies show that individuals’ judgments and choices are often strongly shaped by a reference point. The central idea of this theory is that, when evaluating alternatives, decision makers do not rely solely on the absolute value of an option. Instead, they compare the option against a specific reference point and form a subjective value assessment according to the extent of its relative deviation, which in turn alters the final decision [26]. The source of the reference point is context dependent. It may arise from historical prices, expected returns, experience, social comparison targets, or the way in which a platform presents available options. Therefore, it has a designable nature.

To quantitatively characterize the reference effect, this paper introduces the following mathematical abstraction. Let the set of available alternatives be *S* = {*s*_1_, *s*_2_, …, *s_n_*}. Under the framework of traditional economics, the utility of option *s_i_* depends only on its absolute attributes and is written as *U*(*s_i_*) = *f*(*s_i_*), where *f*(.) denotes the utility function. Under the framework of behavioral economics, if the reference point is set as *r*, then the evaluation of the same option depends on its deviation from the reference point, namely *s_i_* − *r*, and the corresponding reference effect relative utility is given by *U*(*s_i_|r*) = *g*(*s_i_* − *r*). In other words, the introduction of a reference point systematically changes the agent’s subjective valuation of the same option and thereby shifts the final choice outcome.

To illustrate the reference effect more intuitively, this paper uses the classic subscription experiment reported by The Economist [27]. In this experiment, the researchers asked 100 participants to choose among three subscription options. Option A is a web-only subscription at $59 per year. Option B is a print-only subscription at $125 per year. Option C is a bundled subscription including both web and print access at $125 per year. The results show that, when all three options are available, 84 participants choose Option C, 16 choose Option A, and none choose Option B. The researchers then remove Option B and retain only Options A and C. At this point, the choice distribution reverses markedly. The number of participants choosing Option A rises to 68, while the number choosing Option C falls to 32. This phenomenon indicates that, although Option B is not the main target of participants’ choice, it serves as a reference point that strengthens decision makers’ perception of the relative advantage of Option C and thereby reshapes the overall choice structure.

In summary, the introduction of a reference point reshapes individuals’ relative evaluation of option value and ultimately shifts the distribution of group choices. Such a mechanism is widely observed in practical settings, including price presentation, information display, online recommendation, and comparative ranking.

2. Loss aversion

Loss aversion is a core behavioral feature of Prospect Theory. It means that, when individuals evaluate outcomes around a given reference point, the subjective disutility caused by a loss below the reference point is usually greater than the subjective utility brought by an equally sized gain above the reference point [28]. In other words, compared with receiving a gain of a certain amount, individuals are often more sensitive to a loss of the same amount. This idea is first systematically introduced by Kahneman and Tversky in Prospect Theory and is further developed by Tversky and Kahneman in Cumulative Prospect Theory [29].

**Figure 1:**
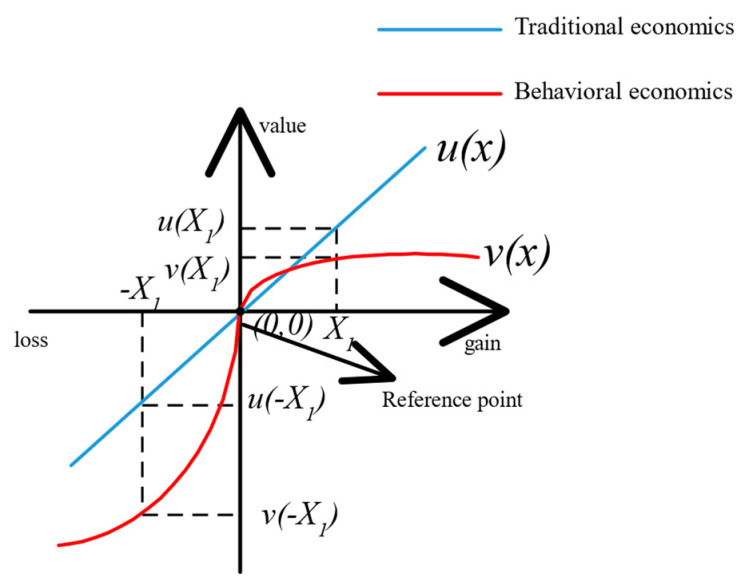
Loss-aversion value function.

Mathematically, this paper characterizes loss aversion by using a piecewise value function defined relative to the reference point. Let *r* denote the reference point and let *u* denote the participant’s actual payoff. Then, the outcome deviation relative to the reference point is written as *x* = *u* − *r*. Here, x≥0 means that the outcome is above the reference point, which corresponds to the gain domain, whereas x<0 means that the outcome is below the reference point, which corresponds to the loss domain. The individual’s subjective value function is defined as

(1) $$U(x)=\left\{\begin{array}{l} x,\;\;\;\;x\geq 0\\ -\lambda x,\;x<0 \end{array}\right.$$

where λ>1 is the loss-aversion coefficient, which captures the amplified sensitivity of individuals in the loss domain relative to the gain domain. When x<0, a negative deviation of a given magnitude causes a stronger change in subjective utility, which indicates that individuals perceive losses more strongly than gains. As shown in Figure 1, when the reference point is set to 0, the value function remains steeper in the loss domain than in the gain domain $\left|U(x_{1})\right|<\left|U(-x_{1})\right|$.

3. Operant Conditioning Theory

**Figure 2:**
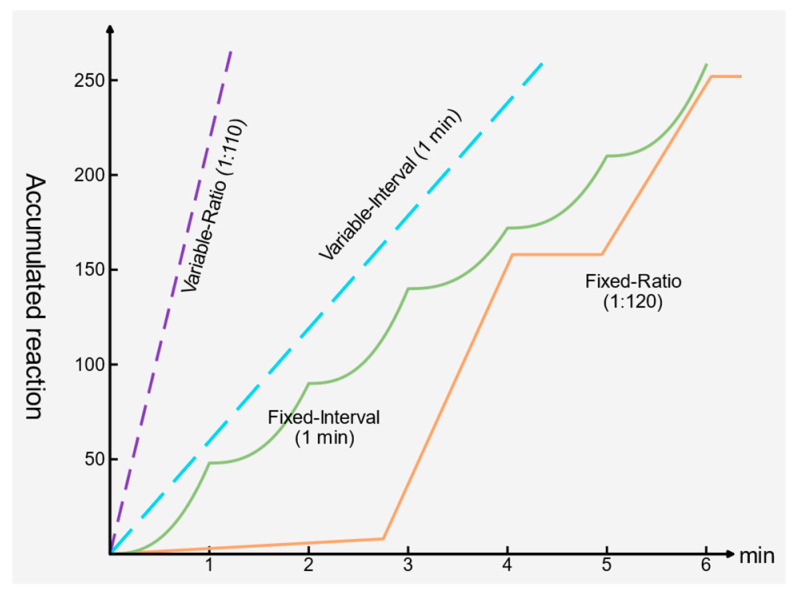
Reinforcement schedules and behavior patterns.

Operant Conditioning Theory holds that individual behavior is continuously shaped by its consequences. When a behavior is followed by a rewarding outcome, the probability that this behavior occurs again in the future increases [30]. With respect to the question of when rewards should be delivered, the reinforcement schedule serves as a key mechanism for behavior shaping. Among these schedules, intermittent reinforcement means that reinforcement is not provided immediately after every target behavior but is delivered at intervals according to a specific rule. Compared with continuous reinforcement, intermittent reinforcement is more effective in sustaining the persistence and stability of behavior. Typical intermittent reinforcement schedules mainly include four types, namely Fixed Ratio (FR), Fixed Interval (FI), Variable Ratio (VR), and Variable Interval (VI) [31]. The distinct behavior patterns generated by these four schedules are depicted in Figure 2. FR, namely fixed ratio reinforcement, means that an individual receives one reinforcement after completing a preset number of behaviors. For example, one reward is given after every 10 target behaviors. Under this mechanism, reinforcement has a stable correspondence with the number of behaviors. As a result, individuals can more easily form a definite expectation about the relation between effort and reward, and thus, this schedule usually produces a relatively fast behavioral response rate. However, because the reinforcement rule is predictable, the behavioral pattern is also more likely to become mechanical, and a short pause in responding often appears after reinforcement is received.

FI, namely fixed interval reinforcement, means that reinforcement is delivered to behaviors that satisfy the required condition after a fixed time interval has elapsed. For example, one reward opportunity is provided every 5 min. Under this mechanism, individuals gradually form an expectation about the time interval. Their behavioral response typically shows the following pattern: responses are relatively infrequent immediately after reinforcement, and the response frequency increases progressively as the next reinforcement time approaches, thus producing a typical step-like or scallop-shaped trend. Therefore, FI is more likely to induce time-oriented behavior rather than continuously stable high-frequency behavior.

VR, namely variable ratio reinforcement, also uses the number of behaviors as the basis for reinforcement, but the number of behaviors required for each reinforcement is not fixed and instead varies randomly within a certain range. For example, the system may provide one reinforcement for every three qualified behaviors on average, while allowing the exact reinforcement to point to fluctuate randomly across different rounds. Since individuals cannot accurately predict when the next reinforcement will arrive, they tend to maintain a high and persistent level of behavioral effort to increase the chance of receiving a reward. Compared with FR, VR is more likely to induce a stable, high-frequency, and extinction-resistant behavioral pattern. For this reason, it is widely regarded as one of the most suitable reinforcement schedules for long-term behavior shaping.

VI, namely variable interval reinforcement, means that the time interval between reinforcement opportunities is not fixed but varies randomly within a certain range. Although individuals cannot determine in advance the exact time at which reinforcement will appear, they tend to maintain a certain level of continuous responding to cope with reinforcement opportunities that may arise at any time. Compared with FI, the behavioral pattern induced by VI is usually more stable and is less likely to exhibit a clear temporal clustering effect. Therefore, VI helps sustain relatively stable ongoing behavior.

## 4. Incentive Mechanism Inspired by Behavioral Economics and Behavioral Psychology

**Figure 3:**
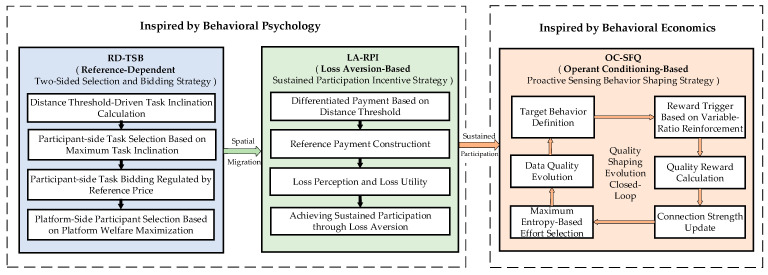
The overview framework of the incentive mechanism inspired by behavioral economics and behavioral psychology.

**Figure 4:**
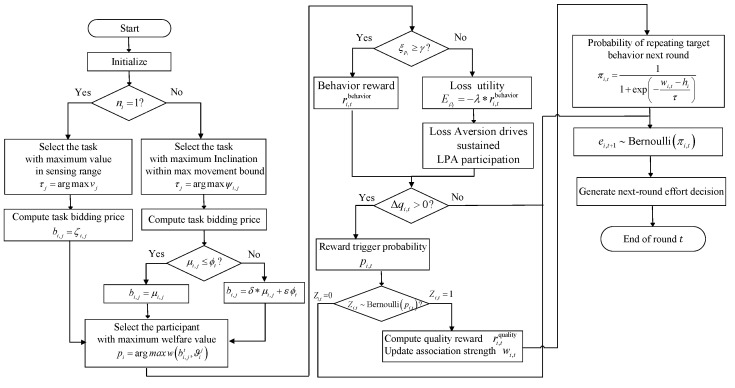
Algorithms flowcharts of the proposed incentive mechanism.

To effectively address the challenges of participant motivation, we construct an integrated incentive framework based on behavioral science, as depicted in Figure 3. This architecture consists of three pivotal mechanisms: RD-TSB, LA-RPI, and OC-SFQ. To facilitate the precise description of these strategies, the key mathematical symbols are outlined in Table 1 for reference. Subsequently, to further clarify the implementation details of this active guidance process, Figure 4 details the algorithmic flowcharts, breaking down the overarching framework into executable computational logics.

### 4.1. Reference Effect-Based Two-Sided Selection and Bidding Strategy (RD-TSB)

#### 4.1.1. Participant-Side Task Selection

This section examines how participants select tasks when multiple alternatives are available under the influence of the reference effect. Based on reference effect theory, a task-selection strategy is designed to align with participants’ behavioral preferences. By carefully configuring reference points, the mechanism guides individuals toward participation decisions that better align with system objectives, thereby jointly improving incentive efficiency and overall system performance. To maximize long-term returns while maintaining spatial balance, the task-selection logic is divided into two dynamically progressive stages according to the number of times a participant has participated.

In the first stage, which corresponds to the initial aggregation period when the participation count $n_{i}$ satisfies $n_{i}=1$, each participant prioritizes sensing tasks located in the HPA where the participant is physically located. In this stage, the participant does not need to move in space, which implies that the movement distance equals 0. The initial task can therefore be completed without incurring any mobility cost.

In the second stage, which corresponds to the reference-driven extension period when the participation count satisfies $n_{i}>1$, the decision scope of a participant expands to the candidate task set $\Gamma_{i,t}$ within the physical upper bound *ϖ*. In this stage, the distance threshold γ acts as a core reference point and reshapes the evaluation structure of task selection. To quantify the psychophysical influence induced by the reference effect, a key metric termed task inclination $\psi_{i,j}$ is introduced. This metric evaluates the candidate tasks and identifies the task with the global maximum inclination $\arg\max\psi_{i,j}$ in the candidate set. The selected task is then added to the task set $S_{i}$, where $S_{i}$ denotes the task set selected by participant $p_{i}$. Unlike traditional models that rely solely on absolute economic utility, the computation of $\psi_{i,j}$ tightly couples the mobility distance with the distance threshold. Its formal definition is given as follows:

(2) $$\psi_{i,j}=\frac{\vartheta_{i}^{j}}{\gamma }+\frac{e^{\eta_{i,j}*\vartheta_{i}^{j}}-1}{v_{j}}$$

In this formulation, $\vartheta_{i}^{j}$ denotes the actual movement distance from participant $p_{i}$ to task $\tau_{j}$, γ denotes the distance threshold set by the platform, $v_{j}$ represents the intrinsic value of the task, the corresponding term $e^{\eta_{i,j}*\vartheta_{i}^{j}}-1$ quantifies the mobility cost, and $\eta_{i,j}$ denotes the willingness coefficient of participant $\rho_{i}$ toward task $\tau_{j}$.

In real vehicular crowd-sensing environments, the sparsity level of participants in LPAs and the backlog of tasks evolve continuously over time. If the distance threshold γ is treated as a static constant, the mechanism cannot respond effectively to sudden supply and demand imbalances. To address this issue, the threshold γ in the task inclination evaluation framework is further extended into an adaptive threshold driven by the supply and demand gap in LPA regions. Its dynamic update rule is defined as follows:

(3) $$\gamma =\max(\gamma_{\min},\min(\gamma_{\max},\gamma_{0}-\iota *\frac{D_{t}-S_{t}}{D_{t}}))$$

In this nonlinear adjustment model, $\gamma_{0}$ denotes the baseline distance threshold of the system. $D_{t}$ and $S_{t}$ denote the total number of tasks that need to be completed in the LPA region at round *t* and the number of tasks that have been completed, which correspond to the absolute demand and the effective supply respectively. The corresponding term $\frac{D_{t}-S_{t}}{D_{t}}$ measures the real-time relative supply demand gap in that region, while ι denotes the gap is sensitivity coefficient. The parameters $\gamma_{\min}$ and $\gamma_{\max}$ provide rigid bounds for the threshold variation and prevent extreme allocation outcomes.

The computation logic in this stage reveals a counterintuitive but practically meaningful causal relationship. Once the distance threshold is introduced, the task preference of participants is influenced by the difference between the distance threshold and the actual distance from the participant to each task. When two tasks provide identical value, the physical movement distance $\vartheta_{i}^{j}$ is strictly proportional to the task inclination $\psi_{i,j}$. This relationship implies that under the psychological reference induced by the threshold γ, a task located farther away, which corresponds to a smaller difference between the mobility distance and the threshold, generates a stronger intrinsic motivation for the participant to select that task $(\vartheta_{i}^{j}-\gamma )\propto {1/\psi }_{i,j}$.

In summary, the introduction of the distance threshold effectively mitigates the inherent tendency of participants to avoid long distance movement. It provides a strong endogenous incentive for participants to move toward LPAs, which fundamentally alleviates the allocation bottleneck caused by spatially imbalance task distributions.

#### 4.1.2. Participant-Side Task Bidding

After completing the distance-threshold-based evaluation of task preference and the spatial-level screening of candidate tasks, participant $p_{i}$ determines the specific bid for the selected task $\tau_{j}$. To regulate and constrain the spontaneous bidding behavior of participants, a second reference point, namely the reference price, is introduced.

First, the platform publishes a public reference price $\phi_{t}$ in round *t*. Since the task value distribution across rounds is assumed to remain relatively stable in this study, this indicator is defined as the average bid of participants who successfully obtain tasks in the previous round (*t* − 1). In addition, to suppress the sharp bid inflation caused by temporary supply and demand fluctuations in individual rounds, the reference price is updated using an exponential smoothing rule, which is formally expressed as

(4) $$\phi_{t}=d\phi_{t-1}+(1-d)\frac{\sum_{p_{i}\in W_{get}^{t-1}}b_{i,j}^{t-1}}{\left|W_{get}^{t-1}\right|}$$

where $W_{get}^{t-1}$ denotes the set of participants selected in round *t* − 1 and *d* in [0.6, 0.9] is the smoothing parameter.

This module is a normative mechanism design component. More specifically, the reference price is introduced to provide a reference-dependent bidding rule and decision constraint at the mechanism-design level. It is not intended to fully capture the complete psychological process underlying participants’ bidding decisions in real-world settings.

Next, participant $\rho_{i}$ evaluates the perceived execution cost of task $\tau_{j}$, the spatial mobility cost $\left(e^{n_{i,j}*\vartheta_{i}^{j}}-1\right)$, and the participant’s historical bid in the previous round. Based on these factors, the participant first computes an initial self-pricing $\mu_{i,j}$:

(5) $$\mu_{i,j}=\zeta_{i,j}+e^{n_{i,j}*\vartheta_{i}^{j}}-1+\beta b_{i}^{t-1}$$

Where $\zeta_{i,j}$ denotes the sensing cost of task $\tau_{j}$ as perceived by participant $\rho_{i}$, $b_{i}^{t-1}$ denotes the historical bid submitted by participant $\rho_{i}$ in round t−1, and $\beta \in \left(0,1\right)$ is a coefficient that adjusts the weight of the historical bid in the pricing process. To ensure variable availability, when the participant does not have a valid historical bid in the previous round, such as during the first participation or when no bid was submitted previously, an initialization rule is applied that sets the historical component equal to the current cost estimate. This treatment ensures that the model remains stable during the initial stage and under sparse participation conditions.

After computing the initial self-pricing value, the participant does not submit it directly. Instead, the participant compares the self-pricing $\mu_{i,j}$ with the publicly announced reference price $\phi_{t}$ and performs a psychological and economic adjustment. When the self-pricing value does not exceed the reference threshold, the bid is considered competitive in the current round, and the participant directly submits $\mu_{i,j}$ as the final bid. When the self-pricing value exceeds the reference level, the participant anticipates a higher risk of rejection due to excessive bid inflation. Under the constraint of the reference price, the participant therefore reduces the expected profit margin and lowers the submitted bid. In this case, the final bid becomes a weighted compromise between the self-pricing and the reference price, which is expressed as

(6) $$b_{i,j}^{t}=\left\{\begin{array}{cc} \mu_{i,j}, & \mu_{i,j}\leq \phi_{t}\\ \delta \;*\mu_{i,j}+\varepsilon \;\phi_{t}, & \mu_{i,j}>\phi_{t} \end{array}\right.$$

where 0<ε,δ<1. The parameter ε denotes the reference factor that captures the degree to which participants are influenced by the reference price, while δ reflects the retained weight of the participant’s self-pricing.

#### 4.1.3. Platform-Side Participant Selection

After completing the distance-threshold-based evaluation of task preference and the reference-based adaptive bidding process, the operational control of the mechanism shifts from the participant side to the platform side. Once participants submit their bids $b_{i,j}^{t}$ adjusted according to the reference effect, the platform evaluates multiple bidders competing for the same task $\tau_{j}$. To balance economic cost and spatial coverage efficiency, the platform does not rely solely on absolute bid values. Instead, it calculates the platform welfare $w\left(b_{i,j}^{t},\vartheta_{i}^{j}\right)$ that each participant can generate when executing the task.

Platform welfare is strictly defined as the difference between the maximum payment that the platform is willing to provide, namely the intrinsic value of the task $v_{j}$, and the actual bid $b_{i,j}^{t}$ submitted by the participant, evaluated along the mobility distance $\vartheta_{i}^{j}$ required for task execution. Its continuous representation under an integral formulation can be expressed as

(7) $$\begin{array}{cc} w\left(b_{i,j}^{t},\vartheta_{i}^{j}\right) & =\int_{0}^{\vartheta_{i}^{j}}\left(v_{j}-b_{i,j}^{t}\right)d\vartheta_{i}^{j}\\ & =\left(v_{j}-\zeta_{i,j}+1-\beta b_{i,j}^{t-1}\right)\vartheta_{i}^{j}-\frac{e^{\eta_{i,j}*\vartheta_{i}^{j}}-1}{\eta_{i,j}},\mu_{i,j}\leq \phi_{t}\\ & =\left(v_{j}-\delta (\zeta_{i,j}-1+\beta b_{i,j}^{t-1})-\varepsilon \phi_{t}\right)\vartheta_{i}^{j}-\delta \;\frac{e^{\eta_{i,j}\vartheta_{i}^{j}}-1}{\eta_{i,j}},\mu_{i,j}>\phi_{t} \end{array}$$

After computing the welfare contribution of all bidders, the platform strictly follows a welfare maximization rule. Specifically, the participant who produces the largest platform welfare value ($\arg max\;\;w\left(b_{i,j}^{t},\vartheta_{i}^{j}\right)$) is selected as the final executor of task $\tau_{j}$. When participant $\rho_{i}$ is selected, the assignment relationship satisfies $\rho_{i}\in W_{\mathrm{get}}^{t}\subseteq P$, where $W_{\mathrm{get}}^{t}$ denotes the set of participants selected in round *t*.

### 4.2. Loss-Aversion-Based Sustained Participation Incentive Strategy (LA-RPI)

Based on the task allocation results obtained by RD-TSB, this section introduces loss-aversion theory to design a differentiated payment rule. By incorporating additional rewards into the reference point, participants experience a stronger perception of loss when their behavior fails to meet the threshold, which further strengthens their willingness to continue participating in subsequent rounds.

During the RD-TSB stage, the platform already obtains the set of tasks assigned to participant $\rho_{i}$, denoted by $G_{i}$, as well as the corresponding cumulative moving distance (CMD) indicator $\xi_{\rho_{i}}$. The indicator $\xi_{\rho_{i}}$ characterizes the overall mobility exhibited by the participant during multiple rounds of task execution. Based on $\xi_{\rho_{i}}$, the platform designs a differentiated payment rule in the LA-RPI stage according to a distance threshold. When the participant’s overall mobility satisfies $\xi_{\rho_{i}}>\gamma$, the platform pays the sum of the task bids and additionally provides a behavioral reward $r_{i,t}^{\mathrm{behavior}}$. Otherwise, only the sum of task bids is paid. Accordingly, the actual payment received by participant $\rho_{i}$ in round *t* can be expressed as

(8) $$O_{P_{i}}=\sum_{\tau_{j}\in G_{i}}b_{i,j}^{t}+1\;\left[\xi_{p_{i}}\geq \gamma \right]\cdot r_{i,t}^{behavior}$$

where $1\left(\cdot \right)$ denotes the indicator function that equals 1 when the condition holds and 0 otherwise.

Under this mechanism, participants typically regard the payment level that includes the additional behavioral reward as the reference payment. Therefore, the reference payment of participant $\rho_{i}$ can be expressed as

(9) $$R_{\rho_{i}}=\underset{\tau_{j}\in G_{i}}{\sum }b_{i,j}^{t}+r_{i,t}^{\mathrm{behavior}}$$

When $\xi_{\rho_{i}}<\gamma$, the participant cannot obtain the additional behavioral reward. In this case, the actual payment is lower than the reference payment, which generates a perception of loss. To characterize this payment gap, the loss value of participant $\rho_{i}$ is defined as

(10) $$s_{i}=R_{\rho_{i}}-O_{\rho_{i}}$$

According to loss-aversion theory, individuals are generally more sensitive to losses than to gains of the same magnitude. Therefore, for participants who fail to obtain the reward, the loss utility can be further expressed as

(11) $$E_{\rho_{i}}=-\lambda s_{i}=-\lambda \left(R_{\rho_{i}}-O_{\rho_{i}}\right)$$

where λ denotes the loss-aversion coefficient that captures the degree to which participants amplify the perception of loss. In simulation settings, λ=2.25 is adopted according to studies in prospect theory.

When a participant fails to reach the reward-triggering threshold, the participant not only loses the additional behavioral reward but also experiences a significant negative utility. Under the effect of loss aversion, participants tend to avoid such loss states and adjust their behavior in subsequent rounds to increase the probability of satisfying the reward condition. In other words, LA-RPI converts the subjective loss caused by not receiving the reward into a driving force for continued participation and behavioral improvement, thereby reinforcing participants’ willingness to participate in repeated interactions.

### 4.3. Operant Conditioning-Based Proactive Sensing Behavior Shaping Strategy (OC-SFQ)

Although RD-TSB and LA-RPI improve participation willingness and spatial coverage to some extent, they do not directly constrain the effort exerted by participants during task execution. As a result, data quality can still be limited by insufficient effort. To address this issue, an additional module termed Operant-Conditioning Based Shaping for Quality (OC-SFQ) is introduced. This module enhances participants’ tendency to provide high quality data through reinforcement, without modifying the existing task-selection mechanism or the basic payment structure.

The core idea of OC-SFQ is to define data quality improvement as the target behavior and to provide reinforcement rewards according to a variable ratio schedule after an uncertain number of occurrences of the target behavior. At the same time, an error-driven update of associative strength is adopted to characterize the subjective linkage between behavior and outcome. This design forms a closed loop evolution mechanism that connects effort, quality, reinforcement, and subsequent effort.

1. Define target behavior. Let the maximum sensing capability of participant $\rho_{i}$ be denoted by $a_{i}\in \left[0,1\right]$. This parameter represents the upper bound of data quality that the participant can provide under objective constraints such as hardware limitations. The data quality $q_{i,t}$ therefore satisfies $q_{i,t}\leq a_{i}$. In task round *t*, the effort decision of participant $\rho_{i}$ is modeled as a binary variable $e_{i,t}$, where $e_{i,t}=1$ indicates that the participant chooses to increase effort, and $e_{i,t}=0$ indicates that the participant maintains the previous effort level. When the participant chooses to increase effort, the data quality increases by a positive increment Δ>0 relative to the previous round. The evolution of data quality for participant $\rho_{i}$ is then expressed as

(12) $$q_{i,t}=\left(q_{i,t-1}+\Delta \right)e_{i,t}+\left(1-e_{i,t}\right)q_{i,t-1}$$

where $q_{i,t}$ and $q_{i,t-1}$ denote the data quality at the current round and the previous round, respectively.

Furthermore, the actual quality improvement between two consecutive rounds is defined as $\Delta q_{i,t}=q_{i,t}-q_{i,t-1}$. The platform identifies that a target behavior occurs if and only if $\Delta q_{i,t}>0$. In other words, the submission of data that reflects a quality increase induced by higher effort is regarded as the target behavior in this study.

2. Employ a Variable Ratio (VR) reinforcement schedule to trigger rewards. According to operant conditioning theory, VR reinforcement maintains high response rates and provides strong resistance to extinction by providing rewards after an unpredictable number of responses. VR is adopted because OC-SFQ aims to sustain repeated quality-improving behavior over long-term interactions, rather than merely triggering occasional effort increases. Its strong resistance to extinction is particularly important for preserving high-effort behavior when reinforcement becomes less frequent in the maintenance stage. To balance rapid behavior shaping with cost-effective long-term maintenance, OC-SFQ implements the VR mechanism through a dynamic probability model based on Bernoulli trials. Specifically, for each valid target behavior of a participant, the platform triggers a reinforcing reward and resets the ratio segment with a probability $p_{i,t}$; otherwise, the system proceeds directly to the evaluation of the next target behavior.

Formally, we introduce a random variable $Z_{i,t}∼\mathrm{Bernoulli}\left(p_{i,t}\right)$, where $Z_{i,t}=1$ indicates that reinforcement is triggered after the current target behavior, and $Z_{i,t}=0$ indicates otherwise. This implementation is equivalent to defining the number of target behaviors required between two reinforcements, *N*, as a geometric distribution $\mathrm{Pr}\left(N=n\right)={\left(1-p_{i,t}\right)}^{n-1}p_{i,t}$ with an expectation $E\left[N\right]=1/p_{i,t}$. Consequently, the reinforcement density of the system, representing the average required number of responses, is controlled directly by the trigger probability $p_{i,t}$, thereby manifesting the ratio characteristic. The Bernoulli-trial implementation is adopted because it enables state-dependent and continuous adjustment of reinforcement density through the trigger probability $p_{i,t}$. Compared with a traditional count-based implementation, it is more suitable for realizing the transition from early-stage shaping to later-stage low-cost maintenance.

To achieve dynamic adjustment, the reinforcement trigger probability is designed as a piecewise function of the participant state. We first define a stage indicator function $s_{i,t}$ as follows:

(13) $$\;s_{i,t}=\left\{\begin{array}{cc} Formation, & q_{i,t}<a_{i}\\ \mathrm{Maintenance}, & q_{i,t}=a_{i} \end{array}\right.$$

where $a_{i}$ denotes the maximum sensing capability of participant $\rho_{i}$. Accordingly, the update rule for the dynamic probability $p_{i,t}$ is given by the following:

(14) $$p_{i,t}=\left\{\begin{array}{cc} p_{\min}+\left(p_{\max}-p_{\min}\right)\exp\left(-\kappa T_{i,t}\right), & s_{i,t}=Formation\\ p_{\mathrm{VR}}, & s_{i,t}=\mathrm{Maintenance} \end{array}\right.$$

In this formulation, $T_{i,t}$ represents the cumulative number of target behaviors performed by the participant, and κ>0 controls the exponential decay rate of the probability during the shaping phase, where $p_{\mathrm{VR}}$ denotes the baseline reinforcement probability in the maintenance stage. The parameters $p_{\min}$ and $p_{\max}$ define the upper and lower bounds of the probability ($p_{\min}\left[0.05,0.1\right]$, $p_{\max}\left[0.4,0.6\right]$).

This piecewise design offers distinct advantages: during the behavior formation stage, the system provides high-frequency initial reinforcement to accelerate the establishment of the behavior–reward association, while the trigger probability decays smoothly as the number of participations $T_{i,t}$ increases. When the participant’s quality approaches the capability limit and enters the maintenance stage, the system adopts a fixed VR baseline probability $p_{\mathrm{VR}}$ (for instance, $p_{\mathrm{VR}}=0.2$ in a VR5 schedule). This approach maintains stable behavioral output through uncertain reinforcement while effectively reducing the reinforcement frequency and system budget.

3. The calculation of reinforcement rewards and the update of association strength. When a participant performs the target behavior and successfully triggers the reinforcement mechanism (i.e., $\Delta q_{i,t}>0$ and $Z_{i,t}=1$), the platform grants an additional quality reward $r_{i,t}^{\mathrm{quality}}$. This value is determined dynamically based on the system budget θ(to ensure that the utility brought by participants to the platform is maximized and remains non-negative, we impose reasonable constraints on the parameter θ based on the maximum utility theory in economics), the total number of tasks completed by the participant in the LPA region $N_{move}$, and the frequency of data quality improvements $N_{\mathrm{imp}rove}$ achieved during these tasks. Specifically, the reward is quantified as follows:

(15) $$r_{i,t}^{quality}=\frac{N_{\mathrm{imp}rove}}{N_{move}}*0.4\theta$$

To rigorously express the incentive effect acting upon the participant, we define the effective reinforcement magnitude ${\tilde{r}}_{i,t}$ as follows:

(16) $$\;{\tilde{r}}_{i,t}=1\left\{e_{i,t}=1\right\}\cdot Z_{i,t}\cdot r_{i,t}^{\mathrm{quality}}$$

where $1\left\{.\right\}$ denotes the indicator function. This formulation implies that a substantial reinforcement effect occurs only when the participant exerts substantive effort ($e_{i,t}=1$) and the system determines a reward distribution according to the variable ratio mechanism.

To characterize the behavior–consequence association in operant conditioning, we maintain a dynamic association strength state variable $w_{i,t}$ for each participant. This variable represents the subjective expectation of the participant that increasing effort leads to reinforcement. We employ an update mechanism based on prediction error to iteratively calculate this association strength:

(17) $$w_{i,t}=w_{i,t-1}+\alpha \left({\tilde{r}}_{i,t}-w_{i,t-1}\right)$$

In this equation, α represents the learning rate, which reflects the sensitivity of the participant to new feedback information. When the target behavior does not receive reinforcement over a long period (${\tilde{r}}_{i,t}=0$), the update formula naturally degenerates to $w_{i,t}=\left(1-\alpha \right)w_{i,t-1}$. This captures the extinction phenomenon where participation behavior declines due to a lack of positive feedback. Conversely, when the target behavior consistently receives reinforcement, $w_{i,t}$ gradually increases, thereby continuously consolidating the willingness of the participant to exert effort.

4. The probability of repeated target behavior and action selection is determined based on the principle of maximum entropy. Although the association strength increases at the cognitive level, this does not imply that the participant takes purely deterministic actions in the physical environment. To characterize the objective trade-off between pursuing expected returns and maintaining behavioral uncertainty, we introduce the maximum entropy principle to construct a stochastic decision policy.

Let *h_i_* denote the objective cost for a participant to increase their effort level, such as the expenditure of additional sensing resources or computational power, and let τ>0 represent the policy temperature parameter. We define the single-step subjective utility for the binary action of the participant as follows:

(18) $$U_{i,t}\left(1\right)=w_{i,t}-h_{i},\;\;\;\;\;U_{i,t}\left(0\right)=0$$

Under the objective of entropy regularization, the probability $\pi_{i,t}$ that the participant decides to repeat the target behavior in the next round, specifically choosing $e_{i,t+1}=1$, is given by the following:

(19) $$\pi_{i,t}=\mathrm{Pr}\left(e_{i,t+1}=1\right)=\frac{1}{1+\exp\left(-\frac{w_{i,t}-h_{i}}{\tau }\right)}$$

Subsequently, the participant executes the actual binary effort choice according to this probability. Each physical action in every round is equivalent to sampling from a Bernoulli distribution:

(20) $$e_{i,t+1}∼\mathrm{Bernoulli}\left(\pi_{i,t}\right)$$

OC-SFQ is a behavior-shaping computational mechanism inspired by operant conditioning theory, whose design objective is to characterize, at the mechanism level, the dynamic relationship among reinforcement, repeated behavioral tendency, and quality improvement.

It should be noted that the key behavioral parameters adopted in this paper are introduced in a theory-informed, rather than strictly psychometric, sense. In particular, the loss-aversion coefficient λ follows the commonly used empirical setting in prospect-theory-related studies, to reflect the stronger sensitivity of individuals to losses than to gains of the same magnitude. By contrast, the learning-rate parameter α is used to characterize the sensitivity of participants to new reinforcement feedback and the update speed of the behavior–reinforcement association. Additionally, while this study seeks to capture behaviorally significant dynamic trends in a computable form, it cannot fully cover the richness and complexity of real psychological processes.

## 5. Experimental Results and Analysis

### 5.1. Simulation Settings

**Figure 5:**
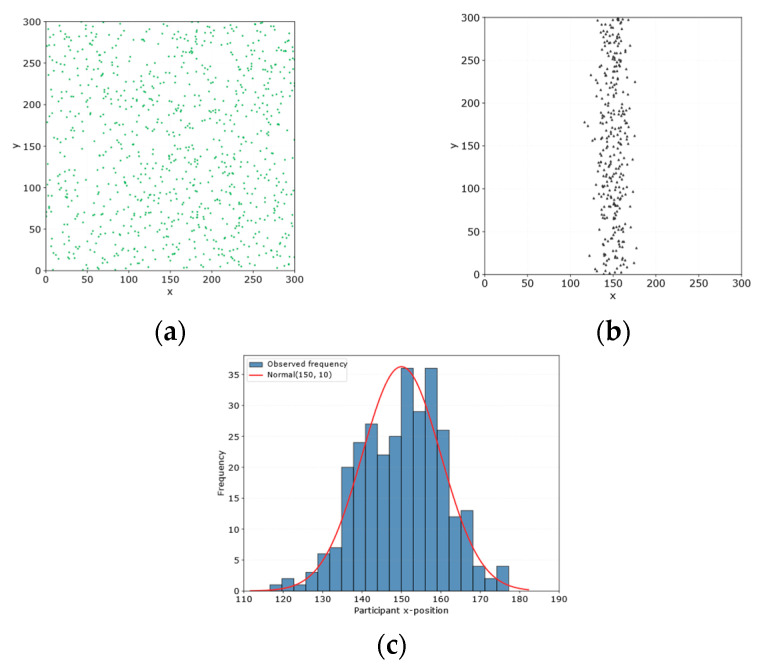
Initial spatial distribution of participants and tasks. (**a**) Initial locations of participants; (**b**) initial locations of tasks; (**c**) coordinate distribution of participants along the *x*-axis.

The simulation experiments are conducted in a sensing area of 300 m × 300 m. A total of 900 tasks is deployed and uniformly distributed over the area. At the same time, 300 participants are initially concentrated in the HPA region. The sensing radius of each task is set to 20 m, and the maximum movement distance of each participant is set to 70 m. The relevant simulation parameters are summarized in Table 2. The spatial distributions of participants and tasks are shown in Figure 3. Specifically, Figure 5a,b presents the initial spatial distributions of participants and tasks in the experimental scenario, respectively, and Figure 5c shows the coordinate distribution of participants along the *x*-axis.

To verify the effectiveness of the proposed method, this paper compares it with MBIM [14] and IBE [16], which were selected because they represent the two comparison paradigms most relevant to this study. Specifically, MBIM serves as a representative baseline for traditional spatial-coverage incentive mechanisms based on mobility-cost compensation and reverse-auction-based mechanism design, while IBE serves as a representative baseline for incentive schemes that incorporate behavioral-economics factors into participant spatial guidance. Therefore, these two baselines provide targeted reference points from the perspectives of traditional mechanism design and behavioral-economics-based incentive design, respectively. Compared with other related methods, MBIM and IBE are more directly aligned with the core problem addressed in this paper, namely spatial guidance under uneven participant distribution and its further extension toward sustained participation and quality shaping.

This section evaluates the proposed mechanism from three aspects, namely participant behavioral migration and task completion distribution, data quality distribution characteristics, and the utility performance of both participants and platform.

### 5.2. Simulation Results and Analysis

#### 5.2.1. Behavior Migration Analysis

**Figure 6:**
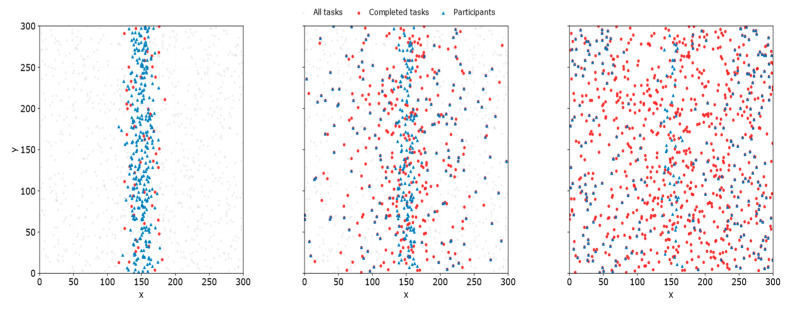
Illustration of participant behavioral migration under the proposed scheme during the initial, intermediate, and final phases, respectively.

**Figure 7:**
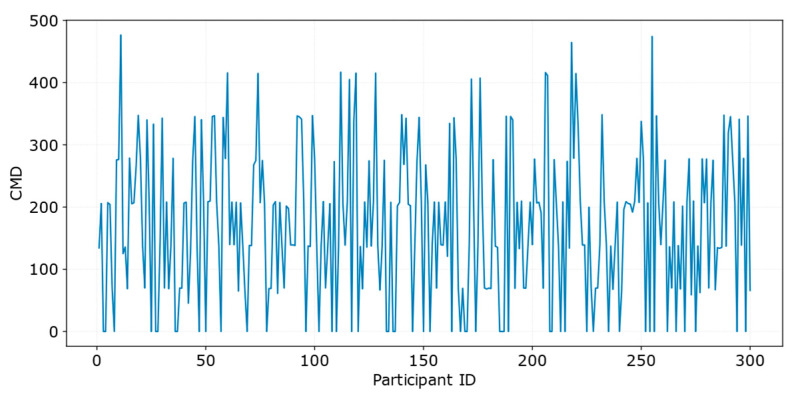
Distribution of CMD for all participants under the proposed scheme.

(1) The behavior-migration and task-completion distributions shown in Figure 6 indicate that the proposed mechanism can effectively drive participants to diffuse from HPA regions toward LPA regions, thereby mitigating the coverage gaps caused by spatial imbalance. Specifically, in the initial state, participants are mainly concentrated within a narrow central band, that is, in HPA regions, whereas tasks are widely distributed throughout the sensing area, leading to a clear spatial mismatch between participant distribution and task distribution. Under the proposed mechanism, the spatial distribution of completed tasks gradually evolves from initially scattered local coverage to a more balanced coverage of the entire area. Meanwhile, the CMD distribution shown in Figure 7 further verifies at the data level that participants indeed undergo actual spatial movement during task execution.

This phenomenon can be mainly attributed to two reasons. First, RD-TSB does not select tasks solely based on the absolute mobility cost. Instead, by introducing a reference-dependent mechanism, it incorporates the relative relationship between the actual movement distance and the distance threshold into task evaluation, thereby increasing the selection probability of LPA tasks that would otherwise be less attractive under a pure cost-minimization criterion. Second, the adaptive distance threshold dynamically adjusts the reference point according to the supply–demand gap in LPA regions, which enhances the mechanism’s ability to perceive and respond to insufficient coverage in sparse areas. Under the combined effect of these two factors, participants are gradually encouraged to migrate from their initial aggregation regions toward under-covered areas, thereby continuously expanding task coverage and improving the overall spatial distribution.

**Figure 8:**
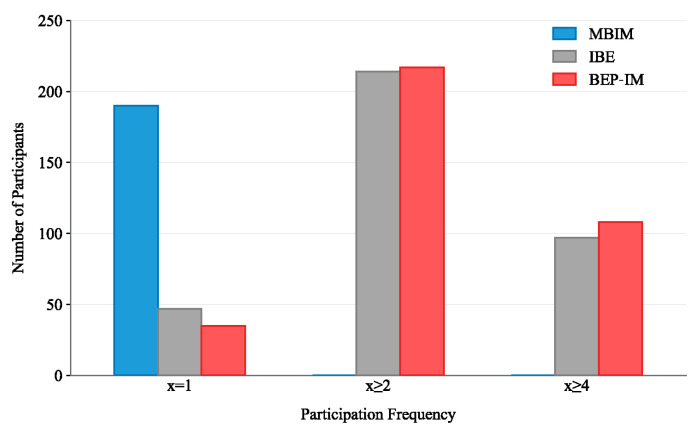
Comparison of participation frequency distribution under different incentive mechanisms.

(2) As shown in Figure 8, the proposed mechanism exhibits a clear advantage in promoting sustained participation in LPA tasks. Specifically, from the distribution of participation frequency, it can be observed that under MBIM, participants are mainly concentrated in the case of participating only once, whereas the number of participants with participation times greater than or equal to 2 is almost zero. By contrast, both IBE and BEP-IM significantly improve the level of sustained participation. Under the criterion of participation times greater than or equal to 2, the number of participants under both mechanisms exceeds 200, with IBE reaching about 215 and BEP-IM about 220. Furthermore, under the stricter criterion of participation times greater than or equal to 4, BEP-IM still outperforms IBE, with IBE at about 97 and BEP-IM at about 108.

This result is mainly attributed to the sustained participation incentive design of LA-RPI in the proposed scheme. Unlike MBIM, which mainly compensates for the mobility cost in a single round, the proposed scheme sets the payment level including the potential behavioral reward as a reference payment. When a participant’s CMD fails to reach the threshold, the participant not only objectively loses the additional reward, but also experiences a subjective sense of loss relative to the reference payment. It is precisely this asymmetric perception of “not obtaining the expected reward” that strengthens participants’ motivation to avoid future loss states, thereby increasing their willingness to continue participating in LPA tasks in subsequent rounds.

#### 5.2.2. Analysis of Positive Sensing Behavior Shaping

**Figure 9:**
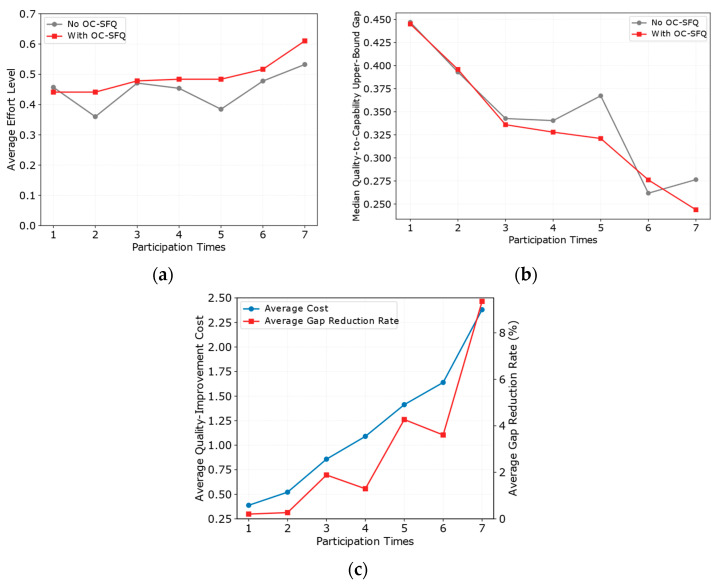
Analysis of participant behavior shaping effects under OC-SFQ. (**a**) Changes in average participant effort level with participation times; (**b**) changes in the median gap between quality and the capability upper bound with participation times; (**c**) changes in average quality improvement cost and average gap reduction rate with participation times.

As shown in Figure 9a–c, the proposed OC-SFQ exhibits clear advantages in improving participants’ effort levels and enhancing data quality. Compared with the No OC-SFQ case, the average effort level under OC-SFQ remains consistently higher, and this advantage becomes more pronounced in the later participation stages. Meanwhile, the median gap between data quality and the upper bound of individual capability remains consistently lower than that of the control group and further converges in the later stages. These observations can be mainly attributed to two factors. First, OC-SFQ defines effortful behavior that produces quality improvement as the target behavior and applies intermittent reinforcement through a variable-ratio schedule, thereby continuously consolidating participants’ effort tendency over repeated interactions. Second, the association-strength updating mechanism accumulates the positive feedback generated by prior reinforcement, enabling participants to gradually form a stable expectation that additional effort can lead to effective returns.

Furthermore, as shown in Figure 9c, although the average cost of quality improvement increases gradually with the number of participations, the average gap-reduction rate continues to rise and reaches its highest value at the seventh participation. This indicates that, even when the marginal cost of quality improvement becomes higher in the later stages, OC-SFQ can still convert reinforcement input into more effective gains in quality convergence. The underlying reason is that the mechanism adopts a strategy of “early-stage reinforcement shaping and later-stage reinforcement maintenance,” which enables the high-effort behavior formed in the early stage to persist, thereby achieving more stable and efficient quality improvement under an acceptable increase in cost.

Overall, Figure 9a–c jointly demonstrates that OC-SFQ can achieve coordinated optimization among effort level, quality improvement, and cost–benefit tradeoff over long-term interactions, thereby validating its effectiveness in shaping high-quality sensing behavior.

#### 5.2.3. Analysis of Cumulative Utility for Participants and the Platform

**Figure 10:**
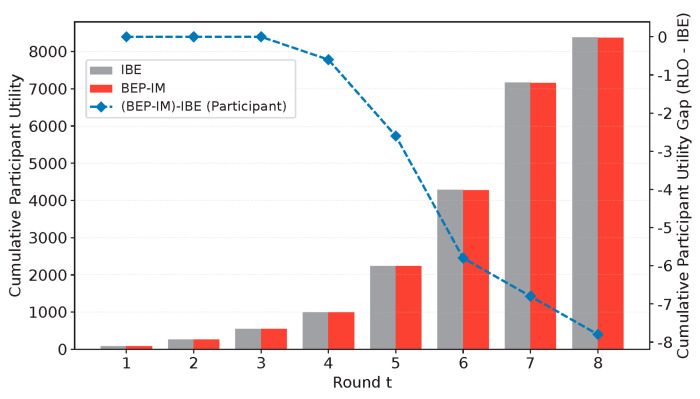
Comparison of cumulative participant utility and utility gap.

**Figure 11:**
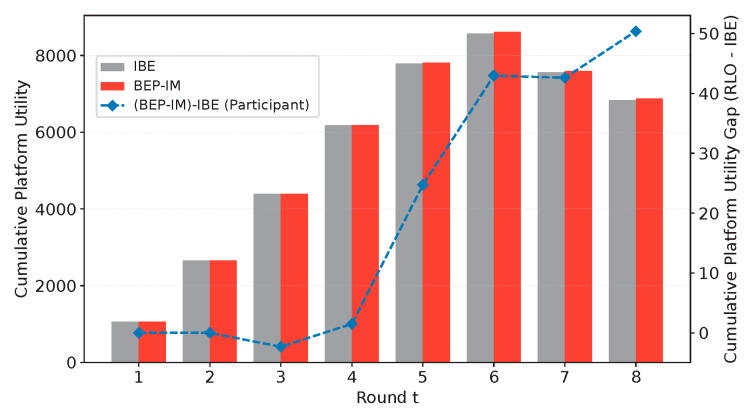
Comparison of cumulative platform utility and utility gap.

The results in Figure 10 and Figure 11 show that BEP-IM exerts utility effects on participants and the platform in different directions over different stages, which reveals a clear stage-dependent pattern. We define the utility of the participant and the platform as $U_{t_{i}}=(O_{\rho_{i}}+r_{i,t}^{quality})-C+E_{\rho_{i}}$ ad $U_{p}=\underset{\rho_{i}\in P}{\sum }\underset{G_{i}\subset \Gamma }{\sum }v_{j}-O_{\rho_{i}}-r_{i,t}^{\mathrm{quality}}$.

First, from the perspective of cumulative participant utility, BEP-IM and IBE remain close in the first few rounds. However, as the number of rounds increases, the gap between them gradually widens, and the value of ((BEP-IM) − IBE (Participant)) remains negative and decreases further in the later stage. This result indicates that, compared with IBE, BEP-IM does not increase participants’ cumulative utility during the experimental period, but instead leads to a slight reduction in their net payoff. The reason is that, while maintaining the original task-execution process, BEP-IM further introduces reinforcement constraints for high-quality behavior, which drives participants to invest more effort or bear higher costs. Although this mechanism helps improve behavioral performance and data quality, it compresses participants’ net utility in the short term.

In contrast, the trend of cumulative platform utility shows that BEP-IM has a stronger advantage in the system-level return. It can be observed that, in the early rounds, the difference in cumulative platform utility between BEP-IM and IBE is relatively small, and, in a few rounds, BEP-IM is even slightly lower than IBE. However, from the middle and later stages onward, the cumulative platform utility under BEP-IM gradually exceeds that under IBE, and the value of (BEP-IM)-IBE (Platform) continues to increase and remains clearly positive in the later stage. This finding suggests that, as the mechanism proceeds over more rounds, the behavioral optimization and quality improvement brought by BEP-IM are gradually converted into more substantial platform gains, so that the platform still obtains higher overall net utility even while bearing additional incentive costs.

Taken together, Figure 10 and Figure 11 show that the role of BEP-IM is not to simply increase the utility of both participants and the platform. Instead, it exhibits a resource-allocation pattern in which a small sacrifice in participants’ local net utility is exchanged for a larger gain in the platform’s overall utility. In other words, while BEP-IM further realizes high-quality behavior guidance, it does not bring a simultaneous increase in participants’ cumulative utility but significantly improves the cumulative net return on the platform side. This result indicates that the proposed mechanism can effectively convert reinforcement incentives into incremental platform gains without substantially increasing the overall payment burden, thereby demonstrating good system-level economic efficiency and mechanism effectiveness.

#### 5.2.4. Analysis of Computational Complexity

To further clarify the practical deployment cost of the proposed mechanism, we briefly analyze the computational complexity of each module. Let *N* denote the number of participants in each round, *M* the number of tasks, $\bar{m}$ the average number of candidate tasks visible to a participant after spatial filtering, $\bar{n}$ the average number of candidate participants for a task, and K the number of finally selected participants. For RD-TSB, the main computational overhead arises from candidate-task construction, task-inclination $\psi_{i,j}$ computation, and platform-welfare evaluation and selection. It’s per-round complexity can therefore be expressed as $O(N\bar{m}+M\bar{n})$. Since the candidate relations are constrained by participant mobility ranges, $\bar{m}\ll M$ and $\bar{n}\ll N$ generally hold, implying that this overhead grows mainly linearly with the scale of effective candidate relations. For LA-RPI, the platform only needs to update the cumulative moving distance, reference payment, and actual payment of the selected participants. Its complexity is therefore mainly related to the number of allocated tasks and can be approximated as $O(K\bar{g})$, where $\bar{g}$ denotes the average number of tasks assigned to each selected participant. For OC-SFQ, each active participant only updates a small number of low-dimensional state variables, such as $q_{i,t}$, $e_{i,t}$, $T_{i,t}$, $p_{i,t}$, $w_{i,t}$, and $\pi_{i,t}$, together with one Bernoulli-trigger operation. Its complexity is therefore O(K). Overall, the additional computational cost of the proposed mechanism is dominated by the candidate-relation evaluation in RD-TSB, whereas the extra overhead introduced by LA-RPI and OC-SFQ is mainly a linear-scale state updating overactive participants, suggesting that the framework remains computationally scalable for round-based platform-side decision making.

From the communication perspective, the platform mainly collects bids, task-execution feedback, and a small amount of state-summary information in each round. Thus, the communication overhead also grows approximately linearly with the number of active participants, without requiring high-frequency continuous interaction.

#### 5.2.5. Parameter Sensitivity Analysis

**Figure 12:**
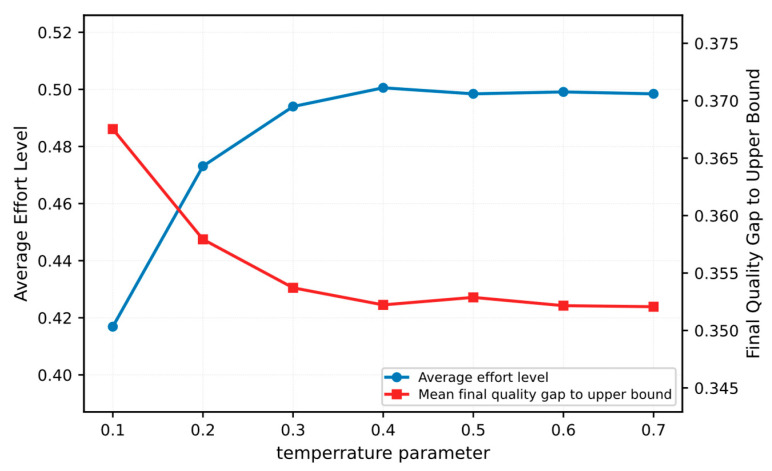
Sensitivity analysis of the policy temperature parameter τ.

**Figure 13:**
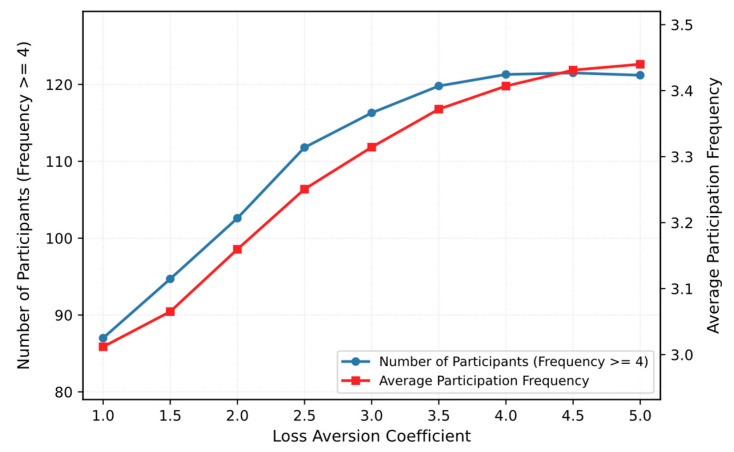
Sensitivity analysis of the loss-aversion coefficient λ.

(1) To evaluate the sensitivity of the proposed mechanism to the policy temperature parameter τ in Equation (19), we further examine how the average effort level and the final quality gap to the individual capability upper bound vary with different values of τ. As shown in Figure 12, when τ increases from 0.1 to 0.4, the average effort level rises steadily, while the mean final quality gap decreases continuously. When τ is further increased to the range of 0.5–0.7, both metrics become relatively stable, with only slight fluctuations. These results indicate that an excessively small τ makes participant decisions overly conservative and limits exploration, which weakens the shaping of high-effort behavior. By contrast, a moderate increase in τ improves the balance between exploration and exploitation, allowing reinforcement feedback to more effectively promote effortful behavior and quality improvement. At the same time, the results also suggest that the benefit of increasing τ becomes saturated beyond a certain range. Therefore, a moderate value of τ, such as τ≈0.4 in our setting, is more suitable for achieving stable effort enhancement and quality convergence.

(2) To further examine the robustness of the proposed mechanism with respect to the loss-aversion coefficient λ, we conduct a sensitivity analysis by varying λ and observing its effect on sustained participation. As shown in Figure 13, both the number of high-frequency participants and the average participation frequency generally increase as λ becomes larger. In particular, the improvement is more pronounced when λ is in the lower range, while the curves gradually flatten after λ reaches a relatively high level, indicating a diminishing marginal gain. This result suggests that a larger λ strengthens participants’ subjective sensitivity to not obtaining the expected reward, thereby enhancing the sustained-participation effect of LA-RPI. At the same time, the saturation trend also implies that the mechanism does not require an excessively large λ to achieve stable improvement. Therefore, the commonly adopted setting λ=2.25 is not only consistent with prospect-theory-related empirical studies, but also lies within a practically effective range in our simulation setting.

## 6. Conclusions and Future Works

To address the dual challenges of imbalanced spatial supply and weak data quality constraints in collaborative sensing for the Internet of Vehicles, this paper constructs a collaborative incentive framework that integrates spatial guidance, participation retention, and proactive sensing behavior shaping.

In the dimension of spatial supply, this paper achieves a Pareto improvement in resource allocation through the synergy of the RD-TSB and LA-RPI mechanisms. The former utilizes a reference-driven utility evaluation to deeply couple task preferences with allocation strategies, spatially guiding participants to migrate toward low-participation areas. The latter strengthens the psychological contract of participants by characterizing asymmetric loss perception, thereby temporally resolving the sporadic nature of task execution in LPAs. The integration of these two mechanisms enables the platform to achieve an efficient and balanced distribution of sensing resources under a limited budget. In the dimension of quality assurance, the OC-SFQ mechanism facilitates a paradigm shift from reactive penalization to proactive shaping. Through the careful design of reinforcement probabilities and dynamic rewards, this mechanism translates high-level effort into a reinforceable target behavior, effectively resolving the collaborative dilemma where participants are physically present but contribute low-quality data.

In future research, the proposed incentive framework can be further extended to align with emerging paradigms in learning-enhanced and edge-intelligent vehicular systems. For instance, integrating communication-efficient data acquisition, online spatio-temporal planning, task offloading, and collaborative inference, as suggested by recent studies on semantic IoV crowdsensing and AI-driven vehicular intelligence [32,33,34], may improve the adaptability and scalability of the mechanism under dynamic communication and computation constraints. Moreover, with the growing availability of crowd-sourced feedback for reinforcement learning alignment [35], incorporating multi-source and feedback-aware reward-calibration strategies may help maintain long-term stability, robustness, and practical deployability in real-world crowdsensing environments.

## Figures and Tables

**Table 1:** Table of symbols.

Symbol	Meaning
x	outcome deviation relative to the reference point
*n_i_*	participation count of participant *i*
*Γ_i,t_*	the candidate task set of participant *i* at round *t*
*ϖ*	physical upper movement bound of a participant
$\psi_{i,j}$	task inclination of participant *i* toward task *j*
$\vartheta_{i}^{j}$	the actual movement distance from participant $\rho_{i}$ to task $\tau_{j}$
γ	the distance threshold
$v_{j}$	the intrinsic value of the task
$\eta_{i,j}$	the willingness coefficient of participant $\rho_{i}$ toward task $\tau_{j}$
*D_t_*	the total required tasks in the LPA region at round *t*
*S* *_t_*	the completed tasks in the LPA region at round *t*
ι	the gap sensitivity coefficient
$\phi_{t}$	a public reference price in round *t*
$b_{i,j}^{t}$	the bid pricing submitted by participant *i* for task *j* at round *t*.
*d*	the smoothing parameter
$\mu_{i,j}$	the self-pricing of participant *i* for task *j*
$\zeta_{i,j}$	the sensing cost of participant *i* for performing task *j*
$w\left(b_{i,j}^{t},\vartheta_{i}^{j}\right)$	the platform welfare
$O_{P_{i}}$	the actual payment received by participant $\rho_{i}$ in round *t*
$\xi_{\rho_{i}}$	the sum of participants’ cumulative moving distances
$r_{i,t}^{\mathrm{behavior}}$	the behavioral reward of participant *i*
$R_{\rho_{i}}$	the reference payment of participant $\rho_{i}$
$s_{i}$	the loss value of participant $\rho_{i}$
$E_{\rho_{i}}$	the loss utility of participant $\rho_{i}$
$a_{i}$	the maximum sensing capability of participant $\rho_{i}$
$q_{i,t}$	the data quality of participant $\rho_{i}$
$e_{i,t}$	the effort decision of participant $\rho_{i}$
$p_{i,t}$	VR reinforcement trigger probability for participant *i* at round *t*
$Z_{i,t}$	Bernoulli indicator of whether reinforcement is triggered.
*N*	number of target behaviors between two reinforcements
$s_{i,t}$	stage indicator for formation or maintenance
$T_{i,t}$	the number of target behaviors performed by participant *i*
$r_{i,t}^{\mathrm{quality}}$	the quality reward of participant *i*
${\tilde{r}}_{i,t}$	the effective reinforcement magnitude
$w_{i,t}$	The association strength state variable
*h_i_*	the objective cost for a participant to increase effort level
$\pi_{i,t}$	probability that participant *i* repeats the target behavior in the next round

**Table 2:** Simulation parameters.

Parameter	Value
Participant position on the *x*-axis	x~N (150,10)
Participant position on the *y*-axis	y~U (0,300)
Task value	[20,25]
Participation cost	[3,5]
Willingness coefficient	[0.01,0.03]
Effort-increase cost	$c_{i}$~U (0.1,0.3)
Gap sensitivity coefficient	[40,160]
Baseline distance threshold	200
Minimum distance threshold	100
Maximum distance threshold	300

## Data Availability

Dataset available on request from the authors.

## Appendix A

In practical vehicular crowdsensing scenarios, HPAs and LPAs are not merely literal labels of high or low participation density, but usually correspond to road regions with different vehicle-arrival intensity and local sensing supply–demand conditions. Specifically, HPAs typically include urban centers, arterial roads, major intersections, or hotspot road segments during peak periods, where vehicle arrivals are relatively dense and local sensing supply can generally satisfy task demand. By contrast, LPAs generally correspond to peripheral roads, suburban branch roads, remote road segments, or off-peak road areas, where vehicle arrivals are relatively sparse and local sensing supply is often insufficient relative to task demand, making these regions more vulnerable to task backlog and sensing coverage deficits.

To make this distinction more operational, the sensing area is partitioned into a set of road-region units w. For region w at round t, let $C_{w}^{t}$ denote the number of active participants, where an active participant refers to a participant that is available in the current round and can reach at least one task in region w within its maximum feasible movement range. Let $A_{w}$ denote the region size and let $B_{w}^{t}$ denote the required number of sensing tasks in that region. Based on these quantities, the local active-participant density is defined as

(A1) $$\sigma_{w}^{t}=C_{w}^{t}/A_{w},$$

and the local supply-demand matching index is defined as

(A2) $$\chi_{w}^{t}=C_{w}^{t}/B_{w}^{t}.$$

Accordingly, a region is classified as an HPA if $\sigma_{w}^{t}\geq \sigma_{high}\;and\;\;\chi_{w}^{t}\geq 1$ and as an LPA if $\sigma_{w}^{t}\leq \sigma_{\mathrm{low}}\;and\;\;\chi_{w}^{t}<1$. Here, $\sigma_{\mathrm{high}}$ and $\sigma_{\mathrm{low}}$ can be set as an operational criterion, for example, using the 75th and 25th percentiles of the regional active-participant-density distribution in the current round, respectively.
